# Effect of Acute Physical Exercise on Executive Functions and Emotional Recognition: Analysis of Moderate to High Intensity in Young Adults

**DOI:** 10.3389/fpsyg.2019.02774

**Published:** 2019-12-20

**Authors:** Haney Aguirre-Loaiza, Jaime Arenas, Ianelleen Arias, Alejandra Franco-Jímenez, Sergio Barbosa-Granados, Santiago Ramos-Bermúdez, Federico Ayala-Zuluaga, César Núñez, Alexandre García-Mas

**Affiliations:** ^1^Department of Psychology, Catholic University of Pereira, Pereira, Colombia; ^2^Physical Education, University of Quindío, Armenia, Colombia; ^3^Research Group Physical Activity, Cumanday, Manizales, Colombia; ^4^Psychology, Cooperative University of Colombia, Pereira, Colombia; ^5^Department of Physical Action, Caldas University, Manizales, Colombia; ^6^Psychology Program, Universidad de Medellín, Medellín, Colombia; ^7^Department of Basic Psychology, University of the Balearic Islands, Palma, Spain

**Keywords:** physical exercise, cognitive neuroscience, cognitive performance, executive functions, emotional recognition, exercise psychology

## Abstract

Physical exercise (PE) is associated with cognitive changes and brain function. However, it is required to clarify the effect of PE in different intensities, population groups conditions and the EF duration over different cognitive domains. Besides, no studies are known to have evaluated the contextual emotional recognition. Therefore, we studied the effect of acute PE of moderate intensities up to higher ones to the executive functions and the contextual emotional recognition. The participants were evaluated and classified in two experiments according to the IPAQ short form self-report and control measures. In both experiments, the groups were randomized, controlled, and exposed to one session of indoor cycling through intervals of high measure intensity (75–85% HRmax). Experiment 1 comprised young adults who were physically active (PA) and healthy, apparently (*n* = 54, *M*_age_ = 20.7, *SD* = 2.5). Experiment 2 involved young adults who were physically inactive (IP) and healthy, apparently (*n* = 36, *M*_age_ = 21.6, *SD* = 1.8). The duration was the only factor that varied: 45 min for PA and 30 min for PI. The executive functions were evaluated by the Stroop, TMT A/B, and verbal fluency, and the emotional recognition through a task that includes body and facial emotions in context, simultaneously. The analysis of factorial mixed ANOVA showed effects on the right choices of the indoor cycling groups in the PA, and the time response in PI. Also, other effects were observed in the controlled groups. TMT-A/B measures showed changes in the pre-test–post-test measures for both experiments. Verbal fluency performance favored the control group in both experiments. Meanwhile, the emotional recognition showed an effect of the PE in error-reduction and enhanced the scores in the right choices of body emotions. These results suggest that the EF with intensities favored cognitive processes such as inhibitory control and emotional recognition in context. We took into account the importance of high-complexity tasks design that avoid a ceiling effect. This study is the first on reporting a positive effect of PE over the emotional contextual recognition. Important clinical and educational implications are presented implications which highlight the modulatory role of EF with moderate to high intensities.

## Introduction

Physical exercise (PE) is an important environmental factor with positive effects on the brain and healthy behavior along different life stages (Herting et al., 2014; Hillman et al., 2014; Etnier et al., 2019), mainly when considering that less than 60% of world population does not do required PE (World Health Organization [WHO], 2018, World Health Organization [WHO], 2007). Such data, nowadays, are increasing and associated with sedentary lifestyles and low PE involvement (Guthold et al., 2018; Päivärinne et al., 2018). Thus, PE is considered a non-pharmacological strategy that has direct effects in functional and cognitive brain structures (Herting et al., 2014; Erickson et al., 2015; Prakash et al., 2015; Voss et al., 2015; Hagger, 2019). Researchers have addressed the questionings of the effect of EF on cognition from two perspectives. On the one hand, chronic effects in PE (e.g., weeks, months, and/or years). On the other hand, the immediate effect of the acute PE (e.g., one session). This study focuses on the second perspective, treating the effect of acute PE on cognitive processes and emotional recognition.

Most of the reviewed literature matches on observing positive changes of cognition after PE (Reed and Ones, 2006; Lambourne and Tomporowski, 2010; Chang et al., 2012; Donnelly et al., 2016; Ludyga et al., 2016; Sáez de Asteasu et al., 2017; Esteban-Cornejo et al., 2019; Etnier et al., 2019; Hillman et al., 2019; McSween et al., 2019). Likewise, it has been demonstrated that an acute PE brings benefits for some ways of performance on cognitive tasks after exercise. By the same token, executive functions improve after PE (Wu et al., 2019). Recent studies, however, affirm that there is not enough evidence that PE is positive for cognition or brain function and structure in child and young samples (Gunnell et al., 2018). What explains these possible contradictions is the huge methodological variety, and therefore, new investigative contributions might clarify and provide evidence of the relation of PE on cognition (Pontifex et al., 2019).

The relation between PE, cognition, brain functions, and structures seem to rest upon several factors (Chang et al., 2012, 2015; Erickson et al., 2015; Gunnell et al., 2018). For instance, duration, intensity, and PE modality, as well as the vital cycle, the type of cognitive performance and physical condition of the participant. Even the effect of PE can vary depending on the cognitive domain (Pontifex et al., 2009, 2019). These factors are relevant in questioning the relation between PE, brain and cognition. Hence, further research is required that identifies its modulating role (Labelle et al., 2013; Ludyga et al., 2016).

Addressing the related factors of PE (e.g., type, intensity, and duration), some contributions have pointed out that aerobic PE can improve cognitive performance in both young and old adults (Chang et al., 2012; Joyce et al., 2014; Tsukamoto et al., 2016). Specifically, Physiological changes have been observed by activating circuits of the prefrontal and occipital cortex in tasks that include a great cognitive effort (e.g., attention and executive control) (Chen and Chang, 2012; Basso et al., 2015). Other studies have reported that aerobic PE can enhance cognitive and neurological function (Armstrong and Welsman, 2007; Herting et al., 2014; Li et al., 2014; Reiter et al., 2015). Likewise, aerobic PE is associated with improved memory efficiency (Herting and Nagel, 2013; Voss et al., 2019) and inhibitory control performance (Pontifex et al., 2019). Even participants who were exposed to the multimodal combination (training of aerobic PE followed by cognitive training) showed improvement in cognitive performance as compared to those who only had a cognitive training (Wang et al., 2019a).

The immediate effects of PE on cognitive performance seem to be conditional on intensity of PE. Inverted U hypothesis (Gutin, 1973) supports that cognitive performance tends to have more benefits after a moderate intensity of aerobic PE (>64% Maximum heart rate – HRmax) in contrast to low ones (<50% HRmax) or high intensities (>80% HRmax). In this regard, the evidence which supports the inverted U hypothesis is wide (Pontifex et al., 2009; Hillman et al., 2014; Li et al., 2014; De Souto Barreto et al., 2016; Ludyga et al., 2016). Nonetheless, current studies inform that high intensities of PE allow to have benefits in cognitive domains as inhibitory control (Quintero et al., 2018), memory and metacognition (Zuniga et al., 2019). Further, it is important to underscore that the number of studies of PE interventions of moderate intensity is actually small compared to interventions of moderate or low intensities (Browne et al., 2017). There are even more limited studies that address the transition of moderate and high intensities.

As a matter of fact, there is no agreement on the effect of duration, type or intensity of PE on cognition and the different types of cognitive domains (Vazou et al., 2019). A line of research suggested by other authors consists of clarifying the effects of PE in people who are physically active (PA), inactive and/or sedentary. Moreover, it associates more cognitive domains (Sink et al., 2015; Tsukamoto et al., 2016; Vieira et al., 2016; Browne et al., 2017; Peruyero et al., 2017; Silva et al., 2019). Based on the above-mentioned, narrative, systematic, and meta-analytic revisions have suggested an improvement of cognitive performance after an acute session of PE (Lambourne and Tomporowski, 2010; Chang et al., 2012; Ludyga et al., 2016; Basso and Suzuki, 2017; McSween et al., 2019); yet, constructs such as emotional recognition must be studied in relation to acute PE.

In that way, understanding how environmental factors, such as PE, contribute to the performance of emotional recognition in context, and in ecological situations, is a novel area that answers to current questionings of social and cognitive neuroscience (Aviezer et al., 2008; Kumfor et al., 2018). We assumed that acute PE has positive effects on emotional recognition. Especially, whenever the aerobic exercise is combined with the demands of coordination seems that it gets more benefits on facial emotion recognition (Brand et al., 2019). In fact, the capacity of information processing, which links body movements with body-face recognition (Kumfor et al., 2018), is associated with brain regions such as motor areas, cerebellum, and fusiform gyrus (Committeri et al., 2007; Yogev-Seligmann et al., 2008), that are connected with the acute PE (Li et al., 2014; Esteban-Cornejo et al., 2019; Won et al., 2019). Recent paradigms on emotional recognition have located body information as contextual keys, taking away attention from facial recognition processing (Aviezer et al., 2008, 2012).

Based on the described evidence, that the dose-response relation in terms of intensity, duration and the type of acute PE over cognitive performance, specifically with executive functional tasks and emotional recognition, is an issue that needs to be studied. By solving the previous matter, we would contribute with knowledge about the strategy of PE, which can be a potent and promising environmental source that allows to adjust cognitive processes (executive performance to be precise) and emotional recognition. Among other scopes, it would facilitate the capacity of social human interactions in clinical and academic activities that improve the mental health of people. Thus, the main objective is to study the effect of acute PE of indoor cycling with a range of moderate and high intensity (75–85% HRmax) in regard to executive functions and emotional recognition in PA and inactive people and high apparently.

## Materials and Methods

### Design and Participants

**FIGURE 1:**
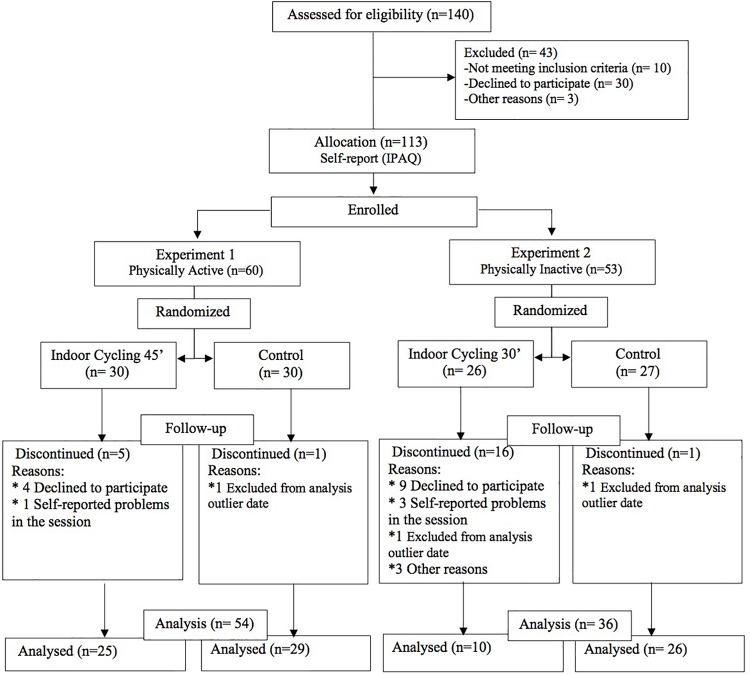
Flow of participants throughout the study.

Two experiments were designed, controlled and randomized with pre-test–post-test measures, for an indoor cycling session. The process of choosing, randomizing, and selecting is shown in Figure 1. Initially, (*n* = 140) were recruited, and 90 university students were analyzed (*M*_age_ = 21.0, *DE* = 2.3). In both experiments, people were randomized by a computer-generated list through Excel (function RANDBETWEEN) from 1 to 60 in PA participants, and between 1 and 53 in physically inactive. The first experiment was carried out in young adults who were PA, and apparently healthy (*n* = 54). La They were randomly assigned to the experimental group and were exposed to a 45 min-session of indoor cycling, whereas the control group had no PE. The second experiment involved physically inactive young adults (*n* = 36) who were randomly selected for the control and experimental groups. The indoor cycling session in the second experiment lasted 30 min. The intensity of a session of PE was moderate-high ranging between 75 and 85% HRmax. The intensity ranges are: moderate between 64 and 76% HRmax, and High 76–96% HRmax (ACSM, 2018) The HRmax was calculated by Karvonen et al. (1957) 220-age (see section “Procedure”).

### Measure

#### General Characteristics

The participants answered a battery of instruments according to pre-experimental protocol (see section “Procedure”). Table 1 provides data such as age, body mass, resting heart rate (supine position for 10 min), cognitive screening (*MoCA*), self-report of physical activity (*IAPQ*), and Depression (*BID*). As expected, significative differences in the IPAQ self-report were observed (*p* < 0.000).

**TABLE 1:** Characteristics of participants.

**Characteristics**	**Physically active (*n* = 54)**		**Physically inactive (*n* = 36)**		***t***	***p***
	**Experiment 1**		**Experiment 2**			
	***M***	***SD***	***M***	***SD***		
Age (years)	20.7	2.5	21.6	1.8	–0.029	0.977
Body mass (kg)	65.5	13.8	64.3	10.6	0.325	0.746
Resting heart rate (bpm)	63.9	11.0	70.8	8.3	–1.83	0.73
Physical activity – IPAQ (METs)	6190.8	5087.1	176.8	195.6	7.07^∗∗^	0.000
Depression – BDI	7.3	5.9	9.94	6.7	–1.89	0.062
Cognitive screening – MoCA	26.57	1.2	26.06	2.2	1.36	0.176

*BPM, Beat per minute; IPAQ, International physical activity questionnaire; METs, Caloric expenditure; BDI, Beck depression inventory; MoCA, Montreal cognitive assessment. ^∗∗^*p* < 0.01.*

#### Measurement Screening

##### Cognitive screening

The MoCA: *Montreal Cognitive Assessment* measures 10 cognitive domains with satisfactory results of sensitivity and specificity, which fluctuate between 82–90% and 75–87%, respectively (Nasreddine et al., 2005). The MoCA is one of the most used instruments in clinics and research that identifies early cognitive changes. Cut-off points vary depending on educational and cultural level (O’Driscoll and Shaikh, 2017). Hence and having in mind Colombian population, one cut-off and exclusion point was assumed <24 (Pedraza et al., 2014, 2016; Gil et al., 2015).

The BDI – II: Beck Depression Inventory tests the existence or severity of depressive symptoms with 21 items. Spanish version was used (Sanz and García-Vera, 2013). The BID-II supports satisfactory psychometric evidence (Wang and Gorenstein, 2013). It was excluded such individuals whose score was >30 were excluded. The observation of depressive symptomatology can constitute a confusing factor, and thereupon, it was relevant to consider this control measure.

##### Physical exercise measure

The IPAQ-SF: The International Physical Activity Questionnaire – Short Form is used to facilitate a measurement and monitoring of physical activity (Craig et al., 2003), and seven questions about the last 7 days in relation to duration, minutes, hours, and days. A total measured score in METs (metabolic equivalent of task minutes per week) is recorded. The three classification levels are the following; low (e.g., walking), moderate (e.g., recreational cycling), and strong (e.g., intense aerobic activity). It is recognized as one of the most used questionnaires to determine AF level (Forsén et al., 2010; Van Poppel et al., 2010). The evidence of consistency and validity of the scores has been reported in several psychometric revisions (Sanda et al., 2017). The prior measurements are in line with the World Health Organization’s global recommendations for physical activity (World Health Organization [WHO], 2007, 2010). IPAQ-SF was used with the aim of classifying active and inactive physically groups.

PARQ & You: The Physical Activity Readiness Questionnaire has been an easy-to-use tool for people between 15 and 69 years of age. The PARQ & You identifies health and cardiovascular problems from seven questions. It is usually applied to apparently healthy individuals, who have the willingness to take part in PE programs (Warburton et al., 2011). A participant’s self-report which was associated with a cardiovascular problem was excluded from the study.

#### Task of Executive Functions

An executive functioning battery was registered, which is composed of three tasks; Stroop, TMT, and Verbal Fluency. These tasks are widely used in executive function measurement (Strauss et al., 2007). Moreover, other revisions have dealt with the PE effect on cognitive processes (Chang et al., 2012; Ludyga et al., 2016; McSween et al., 2019; Pontifex et al., 2019).

(a) Stroop: it evaluates the inhibitory control as executive process and it is associated with function in the anterior cingular cortex. An adapted version of the Stroop test version was performed in pencil and paper format by manipulating two conditions (Florez et al., 2012). In Stroop-A, the person reads written words, and as soon as it is highlighted, he/she must mention the color and avoid saying the word. In the Stroop-B, the evaluator points to the columns of words that are in color, and the individual reads what is written, but when the evaluator says the word “color,” the person should mention the ink’s color without naming the word. In both conditions, 84 stimuli were found. The response and the percentage of the right choices as well as answer time of the execution in both conditions was measured.
(b) The Trail Making Test (TMT) evaluates attention processes, visual scanning, mental processing, and flexibility (Reitan, 1958). It consists of two assessed parts in paper – pencil format. TMT-A deals with the sequence of ascending numbers from 1 to 25. The individuals must match the locked-numbers. The TMT-B is used for the estimation of executive functions (e.g., attention, planning, cognitive flexibility, and response inhibition). The individual has to match the aligned circles randomly by alternating between upward numbers (1–3) and the letters (A–L) (1-A-2-B, etc.). Normally, the TMT-A and the TMT-B are valid and reliable measures (Rabin et al., 2005).
(c) Verbal Fluency (Phonemic and Semantic): It evaluates the capacity to access lexicon and recover semantic information under limited conditions, and its psychometric properties are satisfactory (Borkowski et al., 1967; Strauss et al., 2007). The individual must mention as many words as he/she can, that start with the letter “S” (phonological fluency). Regarding phonological fluency, he must mention as many words as possible in relation to a category or a common interest. We tested the “animals” category. In both tasks, 60 s were timed. Words from the same morphology or proper names were excluded. The total score is the sum of all the right words that are produced in each category.

#### Task Emotional Recognition

**FIGURE 2:**
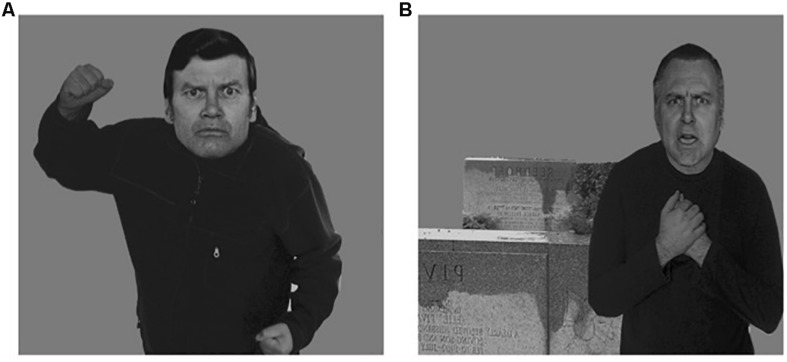
Examples of stimuli of task face-body context. **(A)** Trial congruent face-anger and body-anger. **(B)** Trial incongruent face-anger and body-sadness. Permission obtained from Aviezer et al. (2008).

The experimental task of recognition of body-face emotions was used in context (see Figure 2). This paradigm was originally developed by Aviezer et al. (2008), and used in subsequent studies (Aviezer et al., 2011, 2012; Santamaría-García et al., 2019). This task consists of the representation of four basic emotions (anger, sadness, fear, and displeasure), involving body context. Each emotion is represented by the facial expression along with the body context. In the same way, the facial and corporal emotions can be either congruent or incongruent (e.g., consistent, sadness-face, and sadness-body; inconsistent, sadness-face, and anger-body). We submitted a total of 80 stimuli (Height 11.4 cm × Width 15.2 cm) which were assigned randomly in a 14” computer screen Microsoft Power Point. The task’s command is “You have to select the emotional option that best describes the facial expression.” Each participant had to choose the right answer from six options of basic emotions (sadness, anger, fear, disgust, happiness, and surprise). There is no time limit on task execution. The precision of correct response of the facial emotional expression was scored. The assigned scoring for the correct match of facial emotion =2 points. If the contextual influence matches the body emotion =1 point. When the person neither got the facial emotion, nor the body context right (0 points). The total score turned into a response rate (Response Accuracy = Sum of score/84 × 100).

### Procedure

The volunteers participated in three stages (pre-experimental session, experimental session, and post-experimental session). To maximize transparency and replicability, the indoor Cycling program described in this manuscript follows the Consensus on Exercise Reporting Template (CERT) (Slade et al., 2016) (see Supplementary Table S1).

#### Pre-experimental Session

In the pre-experimental session, the objectives and the scope were presented. In addition to the signature of the informed consent, information about the age, weight, HR at rest, MoCA, BDI-II, IAPQ-SF, and PARQ (see Table 1) was collected. All of these measures were obtained in the days before the indoor cycling PE session. According to the IAPQ-SF results, the participants were classified into one of the two experiments: Experiment 1 – PA volunteers whose physical activity report was moderate and intense. Experiment 2 – physically inactive volunteers whose physical activity was in low category. The participants were instructed regarding clothing, hydration, and feeding before the PE session. The protocol of cognitive measures and emotional recognition from the pretest was executed from 45 up to 60 min before the experimental session in the fitness center. After the evaluation protocol, the instructor informed about the basic concepts, techniques and commands executed in the indoor cycling session. The instructor has a degree in Physical Education with international certification in Indoor Cycle and 10 years of experience in sports training.

#### Experimental Manipulation

Each experiment controlled the HR through Polar-M200, whose HR register is obtained by sensors from the wrist. The intensities of PE were moderates (64–76% HRmax), and high (76–96% HRmax) (ACSM, 2018). Data were gathered through easy connectivity between the device and the computer. The HRmax was calculated by the 220-Age formula. During the experimental session, a research assistant monitored HR behavior and the RPMs that corresponds to the training zone of each participant. The bicycles were standard and relevant to the indoor cycling aims.

Experiment 1 – Physically active group: The participants were randomly assigned to the experimental and control group (see Figure 1). The experimental group performed an indoor cycling session with intensities between 75 and 85% HRmax for 45 min (see Supplementary Figure S1). The PE protocol had three phases. Phase 1, warming up and muscle activation (10 min) 60–65% HRmax between 60 and 90 RPM (revolutions per minute). Phase 2, main work (35 min) which had six sections of 4 min with six peaks of 85% HRmax between 90 and 100 RPM, and active recovery at 75% HRmax. Phase 3, Final recovery (5 min) 60% HRmax between 50 and 70 RPM (See examples of the planimetry – additional material). The group control stayed at rest for 45 min in the facilities of the fitness center. HR measures were the following, HRrest (*M* = 65.4, *DE* = 9.8), HRmean (*M* = 136.8, *DE* = 12.3), HRmax (*M* = 186.1 *DE* = 9.5).

Experiment 2 – Sedentary group: The participants were selected randomly to the experimental and control group (see Figure 1). The experimental group performed an indoor cycling session of 35 min duration with intensities of 75 and 85% HRmax (see Supplementary Figure S1). The protocol had three phases. Phase 1, warming up and muscle activation (7 min) 65–75%HRmax between 70 and 90RPM. Phase 2, main work (17 min) with four sections of 4 min with peaks of 85%HRmax between 50 and 100RPM. Phase 3, final recovery (5 min) 75–65%HRmax between 90 and 70RPM. The control group remained at rest for 30 min after the post-test. HR measures were, HRrest (*M* = 63.1, *DE* = 10.1), HRmean (*M* = 143.1, *DE* = 5.7), HRmax (*M* = 185.4, *DE* = 9.5). In both experimental groups, the musical track was according to the RPM, intensity, intervals, and commands of the instructor (e.g., position three, sitting, etc.).

#### Post-experimental Session

The participants from both experiments had an individual recovery with the monitoring of resting HR. Further, each of them got hydrated during and after PE session. The post-test measure protocols were obtained after getting the resting HR.

### Data Analysis

Both experiments were subjected to the following statistic procedure. The intention-to-treat principle was not carried out, therefore, per-protocol analysis was performed with those who completed the indoor cycling session (see Figure 1). An exploratory analysis of data was executed by calculating tendency central measures (M), of dispersion (SD) and Confidence interval (95% from the mean). *Outliers* that were observed in the box diagram and located below or above from the interquartile range (*Q3–Q1*). In these cases, each datum was analyzed by identifying the information gathering process. To some of them, we applied Winsorizing technique, which consists of adjusting cognitive measures and emotional recognition. Three extreme cases were excluded. Each experiment analysis was executed separately in order to estimate and control the effect of every physical condition (active and inactive). Normality Kolmogorov–Smirnov assumptions were confirmed (*n* > 50) for experiment 1, PA (*p* > 0.05), and Shapiro Wilk (*n* < 50) in experiment 2, physically inactive (*p* > 0.05). The comparison of demographic, cognitive, physical activity, HR, anxiety, and depression variables were estimated with the *T* student test for independent samples. The contrast of the acute PE effect was performed by a Mixed Factorial ANOVA or by partially repeated measures. The dependent variables were treated separately due to the levels and conditions of analysis, which each one of these measures has (e.g., execution time, right choices). Finally, four factorial models were estimated.

(i) The first model was 4 × 2 × 2 for the stroop: four Stroop conditions (Stroop-A right choices, Stroop-A time, Stroop-B right choices, Stroop-B time) × 2 measures (pre-test–post-test) × two groups (Experimental and control).
(ii) The second model 2 × 2 × 2 analyzed TMT measures: two TMT conditions (parts A and B), × two measures (pre-test–post-test) × two groups (Experimental and Control).
(iii) The third model 2 × 2 × 2 analyzed fluency measures with verbal fluency: (phonological “S” and semantic “animals”).
(iv) The fourth model 3 × 2 × 2 analyzed emotional recognition hits: three responses rate (face, body, and errors) × two measures (pre-test – post-test) × two groups (Experimental and Control).

The sphericity of each model was corrected through Greenhouse–Geisser. The meaning of interactions and principal effects were analyzed with the *post hoc* Bonferroni test. The size of the effect was estimated with Eta to the partial square.

### Ethics Statement

We followed the Helsinki’s declaration (WMA, 2013) and the Universal Declaration of Ethic Principles for Psychologist regulations (IUPS, 2008). The evaluation and intervention protocol was approved by the Ethics’ Committee of Health Sciences Department of Universidad de Caldas (CBCS-048 Code, Record 015 of 2017) which is coherent with the rules of the Republic of Colombia (Ministerio de Salud - República de Colombia, 1993). All the participants signed the informed consent acknowledging the purposes, phases, and possible risks of the research. During the phase of experimental manipulation, there were specialized professionals for medical attention if needed.

## Results

Descriptive data of the PA participants (experiment 1) can be observed in Table 2, and for the physically inactive participants (experiment 2) in Table 3.

**TABLE 2:** Descriptive dates physically active (PA) – Experiment 1.

**Variable**	**Indoor cycling PA *n* = 25**				**Control PA *n* = 29**			
	**Pre-test**		**Post-test**		**Pre-test**		**Post-test**	
	***M***	***SD***	***M***	***SD***	***M***	***SD***	***M***	***SD***
**Stroop condition A**								
Accuracy response (%)	95.0	4.0	97.5	2.2	97.1	3.6	97.6	2.4
Response time (seg)	80.4	14.9	73.8	11.4	78.7	24.5	69.4	20.8
**Stroop condition B**								
Accuracy response (%)	98.7	1.9	97.1	8.7	98.6	1.7	99.0	1.5
Response time	73.4	14.3	68.7	11.8	67.0	12.3	58.8	8.8
**Trail making test**								
TMT-A time (seg)	51.6	15.9	36.7	16.2	55.6	21.7	44.0	21.8
TMT-B time (seg)	91.1	43.2	66.1	22.3	90.7	30.6	77.1	27.3
**Verbal fluency**								
Phonemic word “S”	11.6	3.0	13.0	3.4	11.9	3.1	13.9	3.7
Semantic animal	18.6	4.5	19.6	4.3	20.8	4.5	22.0	4.2
**Emotional recognition**								
% Response face	42.3	12.7	44.9	14.5	44.5	11.0	46.1	12.9
% Response body	16.75	10.3	15.4	6.1	18.7	11.5	14.0	5.0
% Error	40.6	12.9	36.2	11.7	39.8	10.9	39.	10.7

**TABLE 3:** Descriptive dates physically inactive (PI) – Experiment 2.

**Variable**	**Indoor cycling PI *n* = 10**				**Control group PI *n* = 26**			
	**Pre-test**		**Post-test**		**Pre-test**		**Post-test**	
	***M***	***SD***	***M***	***SD***	***M***	***SD***	***M***	***SD***
**Stroop condition A**								
Accuracy response (%)	95.1	4.7	98.6	1.4	94.7	7.7	97.8	2.8
Response time (seg)	81.9	19.1	68.2	17.4	70.0	14.8	66.9	12.5
**Stroop condition B**								
Accuracy response (%)	98.2	2.5	98.3	1.6	98.7	1.9	98.9	2.4
Response time (seg)	67.7	16.5	64.0	8.4	67.9	14.5	62.2	16.1
**Trail making test**								
TMT-A time (seg)	46.3	10.9	31.7	10.7	45.7	18.8	38.5	17.4
TMT-B time (seg)	85.6	44.6	73.5	36.9	98.2	47.9	88.2	37.7
**Verbal fluency**								
Phonemic word “S”	9.1	6.1	12.0	2.7	11.8	4.4	17.9	4.4
Semantic animal	20.6	2.9	20.8	3.0	12.1	4.0	19.2	4.8
**Emotional recognition**								
% Response face	48.4	9.7	52.9	14.3	41.2	13.8	41.7	15.0
% Response body	20.8	12.4	16.1	8.1	22.6	9.8	20.9	12.2
% Error	30.9	7.4	31.0	10.2	36.3	13.4	37.3	17.2

### Performance Stroop Task

**FIGURE 3:**
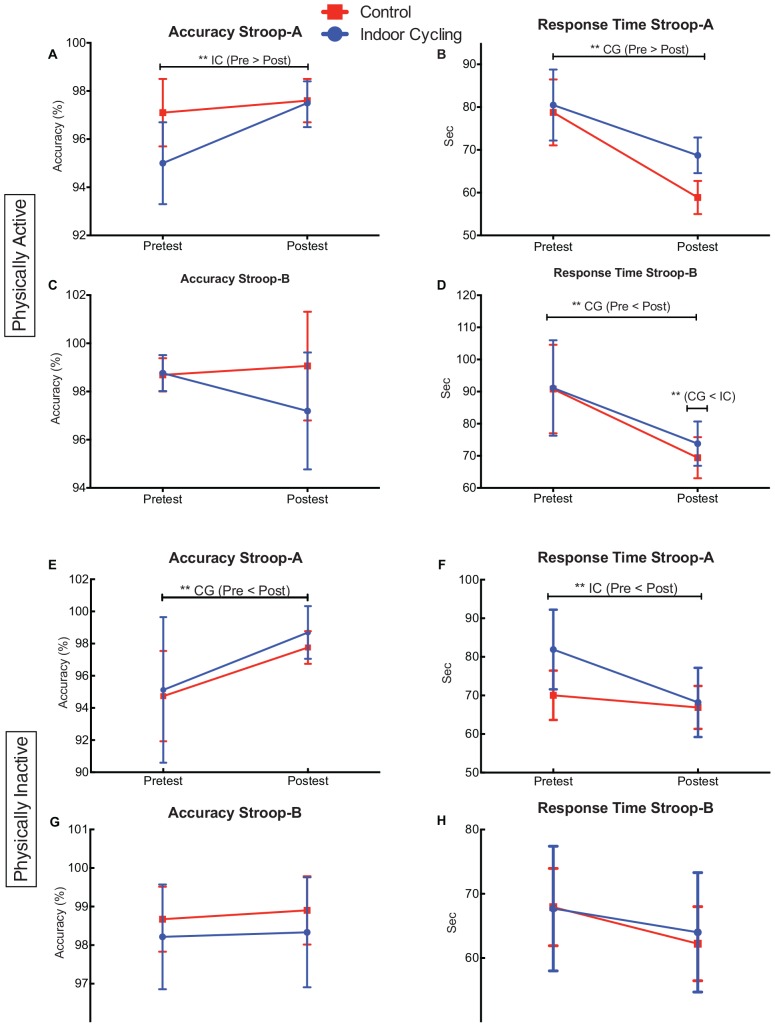
Performance stroop task. Physically active **(A–D)** and physically inactive **(E–H)**. Means and 95% confidence interval of pre-test to post-test of stroop condition **(A,B)**. IC, indoor cycling (Experimental Group); CG, control group. ^∗∗^*p* < 0.01.

The Mix Anova 4 × 2 × 2 (Stroop, time, and group conditions) was not significant in the interaction, [*F*(1.54,80.5) = 452, *p* = 0.588, η*p*^2^ = 0.009]. The stroop and time conditions (pretest and posttest) were significant [*F*(1.54,80.5) = 452, *p* = 0.000, η*p*^2^ = 0.33]. The post-hoc analysis (See Figure 3) showed favorable significant differences for the indoor cycling group in the percentage of right answers of the Stroop-A condition between the pretest and the posttest (*p* = 0.002). In the same way, for the control group, significant changes were observed in the response time of the Stroop-A condition between pretest and posttest (*p* = 0.009), also the answering time of the condition of the Stroop-B, the control group was faster compared to the indoor cycling group (*p* = 0.001).

On the other hand, in the experiment 2 of the PI, the interaction conditions of the stroop, time and group was not significant [*F*(1.00,57.4) = 1.44, *p* = 0.245, η*p*^2^ = 0.041]. The time and stroop conditions showed significant differences [*F*(1.00,57.4) = 5.15, *p* = 0.004, η*p*^2^ =0.121]. The post-hoc median comparison, indicated that the indoor cycling group reduced the answering time of stroop-A between the pretest and posttest (*p* = 0.014). On the other side, the control group showed better right answer percentage of stroop-B between the pretest and posttest (*p* = 0.014) (possibly due to an instrumentation effect).

### Performance TMT-A/B

**FIGURE 4:**
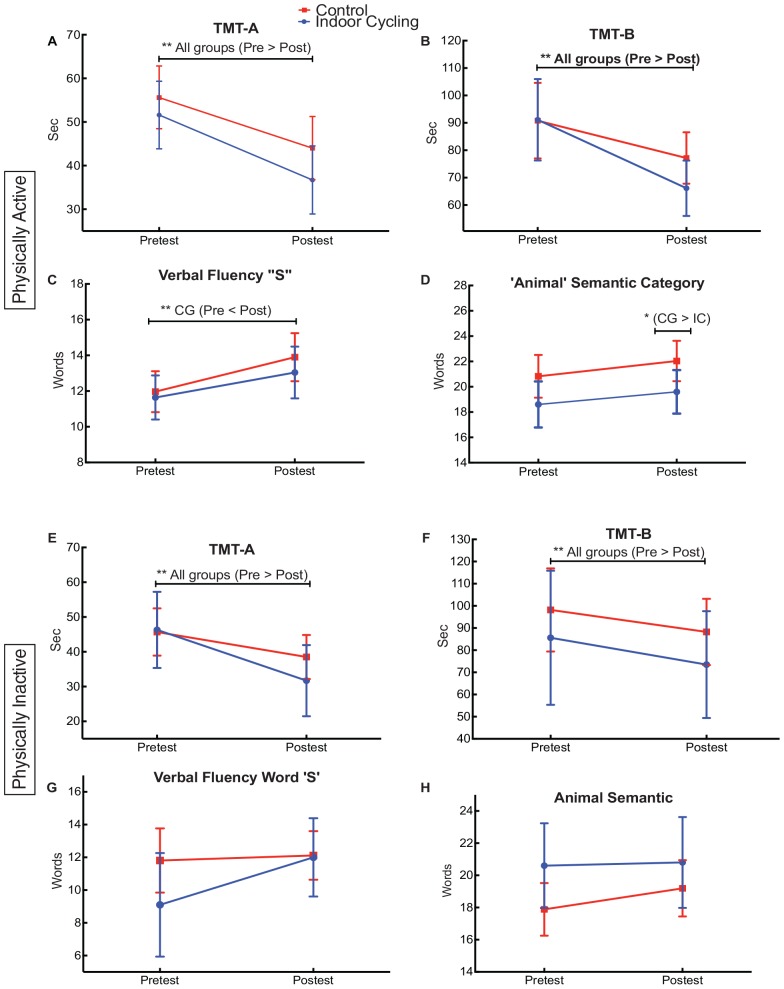
Physically active **(A–D)** and physically inactive **(E–H)**. Means and 95% confidence interval of pre-test to post-test of TMT A/B – verbal fluency. IC, Indoor Cycling (Experimental Group); CG, control group. Physically, ^∗^*p* < 0.05 and ^∗∗^*p* < 0.01.

Task performance of TMT A/B, measures (pre-test and post-test) and the group were not significant [*F*(1.00,52.0) =0.709, *p* = 0.403, η*p*^2^ = 0.013]. Mean comparison indicated that the indoor-cycling group obtained a better average performance than the control group. However, these data were not significant (see Figure 4). The *post hoc* analysis of means Bonferroni comparison showed differences in the pre-test and post-test measures in both TMT-A/B conditions, and in both groups (*p* < 0.000, see Figures 4A,B).

In experiment 2, significant differences in the interaction between TMT-A/B conditions, measures, and the group were identified [*F*(1.00,34.0) =0.199, *p* = 0.658, η*p*^2^ = 0.006]. The interaction between the TMT-A/B conditions and the measures was significant [*F*(1.00,34.0) =6.182, *p* = 0.018, η*p*^2^ = 0.154] (see Figures 4E,F). Although the response time averages were better in the indoor-cycling than the control group, these averages were not significant in both TMT-A/B conditions (*p* < 0.5). The *post hoc* comparison identifies differences of improvement in the execution of time between the pre-test and post-test measures in TMT-A/B conditions (*p* < 0.001, see Figures 4E,F).

### Verbal Fluency

For experiment 1, verbal fluency conditions (greater number of words with the letter “S” and semantic category of animals), measures and the group were not significant [*F*(1,52) =0.057, *p* = 0.812, η*p*^2^ = 0.001]. The *post hoc* analysis indicated that the control group mentioned a greater number of the semantic animals category than the indoor-cycling group in the post-test (*p* < 0.042, see Figure 4D). The group control showed differences in the pre-test and post-test measures in relation to the “S” letter (*p* < 0.010, see Figure 4C).

Interaction between verbal fluency, time (pre-test and post-test) and group was not significant in the physically inactive participants [*F*(1.00,4.0) =6.182, *p* = 0.018, η*p*^2^ = 0.154]. Although the descriptive data show changes in the times, the *post hoc* analysis did not identify.

### Emotional Recognition

**FIGURE 5:**
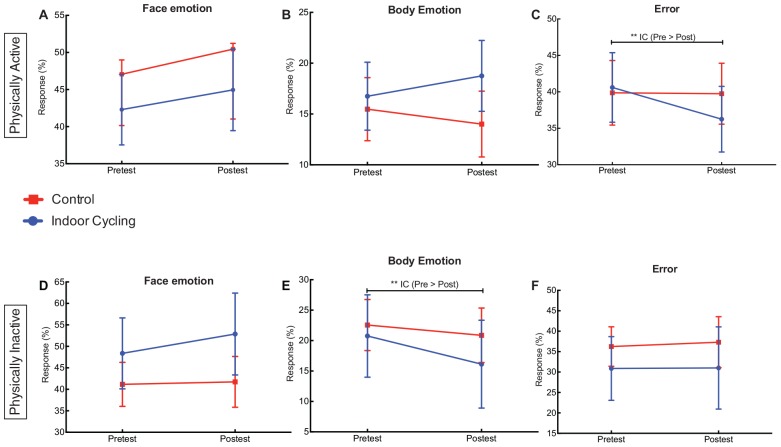
Physically active **(A–C)** and physically inactive **(D–F)**. Means and 95% confidence interval of pre-test to post-test of emotional recognition. IC, indoor cycling (Experimental Group); CG, control group.

Experiment 1 indicated that three emotional recognition conditions (% response face, body and error), measures and the group, were not significant [*F*(1,52) =0.553, *p* = 0.547, η*p*^2^ = 0.011] (Figures 5A,B,F). Measures comparison, with the *post hoc* Bonferroni showed significant differences in pre-test and post-test measures (*p* < 0.32) in error percentage reduction in the indoor cycling group (see, Figure 5C).

For experiment 2, the interaction effect between the responses of emotional recognition, measures and the group was not significant [*F*(1,34) =0.444, *p* = 0.643, η*p*^2^ = 0.013] (Figures 5A,B,F). The main effect of response percentage of emotional recognition in three conditions was significant [*F*(1,34) =8.816, *p* = 0.005, η*p*^2^ = 0.206]. Mean *post hoc* comparison pointed out that the indoor-cycling group had a significant reduction of the percentage of body emotion recognition in pre-test and post-test measures (*p* = 0.028, see Figure 5E). Descriptively, averages indicate that the right choices percentage of facial recognition favored the indoor cycling group (*pre-test, M* = 48.4, *DE* = 9.7; *post-test, M* = 52.9, *DE* = 14.3, see Figure 5D).

## Discussion

Our main objective was to study the effect of acute PE of indoor cycling with a moderate-high intensity range (75–85% HRmax) on executive function, and the emotional recognition in healthy people who are PA and inactive. We have outlined two experiments, each one of them for two conditions, PA (Experiment 1) and inactive (Experiment 2). Overall, we highlight two main results. First, we observed some effects of acute PE on executive functions with a specific PE tendency of indoor-cycling on physically inactive participants. Second, regarding emotional recognition, the group that was exposed to PE indoor-cycling from PA participants (Experiment 1) reduced errors significantly. Similarly, the PE indoor-cycling group from physically inactive participants had a higher percentage of right choices in facial recognition.

These findings provide evidence of the differential effect of demographic conditions (physically active and inactive), and experimental manipulations considering the type, intensity, acute PE, duration on executive functions and emotional recognition. Below, we mention main results.

### Executive Performance and Physical Exercise (Acute PE)

The literature that has dealt with the issues of the effect of acute PE on cognitive processes is wide (Tsukamoto et al., 2016; Akram et al., 2018; Quintero et al., 2018; Leyland et al., 2019; Wang et al., 2019b). In both experiments, we worked with a widely used battery on the executive function evaluation at clinic and research level: Stroop, TMT, and Verbal Fluency.

In this way, Experiment 1 data (PA) indicated that the group, which was subjected to PE indoor cycling, improved significantly in the Stroop-A right choices between pre and post compared to the control group. On the other hand, in the second experiment (physically inactive), the indoor cycling group improved response time in the post-test Stroop-A after the 30-min session (75–85%HRmax). Regarding the control group, Stroop-A right choices in pre-test and post-test measures and response time improved significantly compared to the indoor-cycling group.

In respect of TMT-A/B execution, significant changes enhanced for both groups, which can be explained more by a learning effect of the task than an acute differentiating effect of PE. Regarding phonological and semantic fluency, effects of PE were not observed, but the control group was significantly better. This result must be assumed with reservation on the grounds that this study only presented behavioral measures. Other types of measures and with greater precision can shed light on executive functions and semantic processing. For instance, current contributions point out that the acute PE is associated with greater neuron activation in older adults (Won et al., 2019). The neuron activation and underlying process activation of semantic memory, which is useful for the task performance of semantic processes, which constitutes a promising line of research for future studies (Szepietowska, 2019).

The prior findings are considered from three perspectives. (i) The best performance of the control group, which is PA in the performance of some executive tasks after acute PE, can be explained by a possible physical-cognitive reserve. Thus, optimum cognitive resources would be involved at execution time of the post-test task (Gajewski and Falkenstein, 2015), whereas the indoor cycling group could experiment tiredness due to moderate-high intensities of the HRmax. (ii) From a life cycle perspective, young adults tend to have a relatively stable an executive performance, different from other populations such as children and older adults. Hence, in childhood and old age greater sensibility of executive functions and toward stimuli such as the PE can be evidenced (Ludyga et al., 2016). The prior motive can explain that direct effects are not estimated. (iii) Lastly, the assumption of the hypothesis of the inverted U (Gutin, 1973) indicates that cognitive performance is diminished under high intensities of PE. Some effects of our data could provide evidence to the statement of the inverted U. Another part of our significant outcomes, however, provided evidence that transition of moderate-high intensities has also positive effects on cognitive performance.

Therefore, current studies have reported that high intensities of PE have a positive effect on executive functions in the performance of tasks such as the Stroop and attentional tasks of cancelation (Tsukamoto et al., 2016; Quintero et al., 2018). Additionally, the effect of strong acute PE may last from 30 min to 2 h after EP session (Basso et al., 2015). While it is true that the hypothesis of inverted U has a wide scientific evidence (Pontifex et al., 2009; Chang et al., 2014; De Souto Barreto et al., 2016; Ludyga et al., 2016), the discussion is still open in order to have more solid evidence.

Considering the above-mentioned, studying cognitive processes with great complexity tasks and that are coherent with the postulates of *embodied cognition* sets up a research line to explore. Specifically, simple tasks of cognitive evaluation such as the ones we implemented, and despite being widely used, have some limitations. The ceiling effect has a high rate of right choices with no variability measures, which is characteristic of a healthy neurologically population (McMorris et al., 2016; Mora-Gonzalez et al., 2019). This is a possible cause that justifies why high intensities of PE and healthy population are not sensitive to the performance of simple cognitive tasks.

Thereupon, qualification of tasks with higher complexity, which can underlie motor and cognitive processes, is an important challenge (e.g., Paradigms of Cognitive Motor Interference by using dual tasks). In such way, if the precision with tasks is evaluated, which involve all the body, similar to sport performance, those conditions can generate an intense effect of PE on cognitive processes (McMorris et al., 2016; Browne et al., 2017). In other words, associating tasks of cognitive complexity that involve the body, such as tasks that have an *embodied cognition* perspective, is a methodological and scientific challenge to be explored.

### Emotional Recognition: Hypothesis of Physical Exercise as a Modulator of Social Cognition

In accordance with our revision, the studies that deal with the modulating effect of PE on emotional recognition are limited. In this sense, such issue has been addressed in our study. The found data proved that groups exposed to in one indoor-cycling sessions, from both experiments with moderate-high intensities (HRmax), had an effect on the right choices of emotional recognition in context. Thus, this finding suggests that the acute PE, apart from having cognitive benefits, which were previously mentioned, seems to have a direct effect on recognition emotional processing, and mainly, on tasks that require processing of contextual information.

Studies, which are carried out by magnetic functional resonance (fMRI), have demonstrated that body emotional posture influence in facial expression processing (Poyo-Solanas et al., 2018). Moreover, it has been suggested that the regions that are in charge of processing dynamic body movements are linked to emotional and body recognition (Kumfor et al., 2018). Using tasks of the same kind of our study, emotional recognition patterns in context in teenagers with criminal conduct have been explored (Santamaría-García et al., 2019), and with patients who suffer from frontotemporal dementia (Kumfor et al., 2018). These processes of body-face recognition are related to brain regions, as the fusiform gyrus (Committeri et al., 2007; Yogev-Seligmann et al., 2008), cingulate cortex, superior temporal gyrus, motor areas, and cerebellum. The identification of body and contextual information is very relevant for emotional recognition (De Gelder, 2006; Aviezer et al., 2012; Poyo-Solanas et al., 2018).

To sum up, our results are evidence that supports the modulator role hypothesis of PE in social cognition processes. Also, they let us put in perspective a path for new research lines, which result promising and hopeful so as to enhance the quality of life of people.

### Implications, Limitation, and Future Research Directions

In agreement with the aforementioned, our results have practical implications, which are directly related to the quality of life of people. They are also pertinent for how the real world functions due to the easy access to PE in relation to its cost-benefit and non-invasiveness. The inclusion of programs of health promotion through the PE in school ages, and preventions of diseases in adulthood as a strategy to possible neurodegenerative disease emergence, hence, potential strategies of intervention at a preventive and psychoeducational level (Aguirre-Loaiza et al., 2019). Likewise, instructors and practitioners can train PA and inactive people through intervals of moderate-high intensities as part of a conditioning program as well as parallel purposes that are related with the objective of improving their cognitive processes.

On the other hand, and from a theoretical perspective, our results contribute to the increasing body of knowledge that studies modulator effect comprehension of acute PE on cognitive processes (Pontifex et al., 2019). Additionally, this study considered for the first time the immediate effect of PE in the assessment of emotional-context recognition, its huge scope and potential benefits in social human relationships. In this way, the path for future works in the line of social cognition is marked, modulating the cognitive and brain processes through PE.

This study, however, had some limitations. Firstly, physical activity measures were self-reported. The instruments use, such as the IPAQ-SF in the valoration and allocations to the experiments, was the first limitation. Therefore, more objective evaluations through aerobic conditions will allow a higher control, which is an important criterion for future studies. Secondly, our study kept isolated analysis between two experiments so as to identify a direct effect of duration and PE session intensities. Thus, it would be interesting that both sample conditions are compared (physically active and inactive) in a further analysis. Although it seems that physical condition is not a conditional requirement for temporary improvements and PE benefits (Ludyga et al., 2016). Other works, though, insist on considering physical (Labelle et al., 2013). Finally, our study worked with a single conditional capacity; there are other capacities to be studied (e.g., strength, coordination, among others). Indeed, it is possible the combination of these capacities pose an important work line yet to be studied. Likewise, manipulating a chronic PE program and linking cognitive tasks with complex paradigms is a limitation that if addressed in new proposals, it may clarify of the modulating role of PE. These results should be considered with caution due to the sample size, therefore new work is encouraged (Slade et al., 2016).

After mentioning the limitations, we deem that future studies can address: (i) research that demonstrates the role of EP with different intensities. (ii) Apart from the prior, the assessment and design of other measures of cognitive domains, which are associated to the social cognition and embodied *cognition* with greater complexity, allowing higher sensibility and specificity. For instance, dual tasks in which the body performance is linked. (iii) Effect exploration of other conditional capacities (e.g., coordination). (iv) Motor expertise exploration and analysis of the cognitive-physical reserve relation regarding the cognitive-emotional performance in context.

## Conclusion

In summary, our findings add evidence in which the acute PE with moderate-high intensities of HRmax (75–85%) has positive effects on executive functions, and mainly, in the emotional-contextual recognition.

## Data Availability Statement

The datasets generated for this study are available on request to the corresponding author.

## Ethics Statement

The research was approved by the Ethics Committee of the School of Health Sciences, Caldas University (Code CBCS-048, Acta015 from 2017). All the participants signed the informed agreement and knew the purposes of the investigation and its respective phases. The patients/participants provided their written informed consent to participate in this study.

## Author Contributions

HA-L, JA, IA, AF-J, SR-B, and FA-Z conceived and designed the experiments. JA, IA, SB-G, and H-AL collected data. HA-L and JA performed the statistical analysis. AF-J, CN, SB-G, HA-L, and AG-M interpreted the data. HA-L, AF-J, and SB-G wrote the original draft. HA-L, JA, IA, AF-J, SB-G, SR-B, FA-Z, CN, and AG-M reviewed, edited and drafted the manuscript, and approved the final version.

## Conflict of Interest

The authors declare that the research was conducted in the absence of any commercial or financial relationships that could be construed as a potential conflict of interest.

## Supplementary Material

FIGURE S1 Session Indoor Cycling – Planimetry.

TABLE S1 Consensus on Exercise Reporting Template.
