# Supplementary figures and images for: Effect of Acute Physical Exercise on Executive Functions and Emotional Recognition: Analysis of Moderate to High Intensity in Young Adults
Source: Front Psychol. 2019 Dec 20;10:2774. doi: 10.3389/fpsyg.2019.02774 (PMC6937985; doi:10.3389/fpsyg.2019.02774)

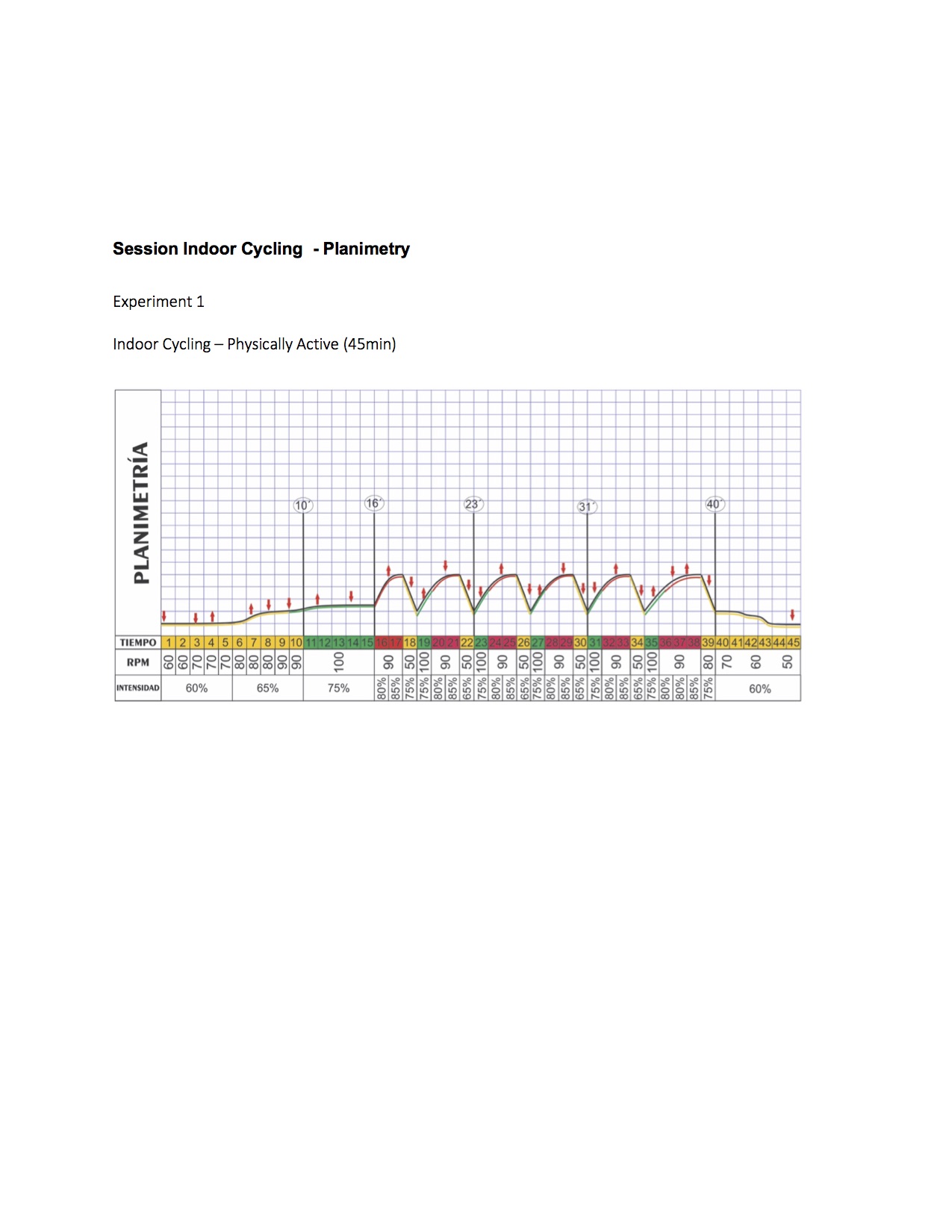

Supplement: FIGURE S1 — Session Indoor Cycling – Planimetry. [file Image_1.jpg]

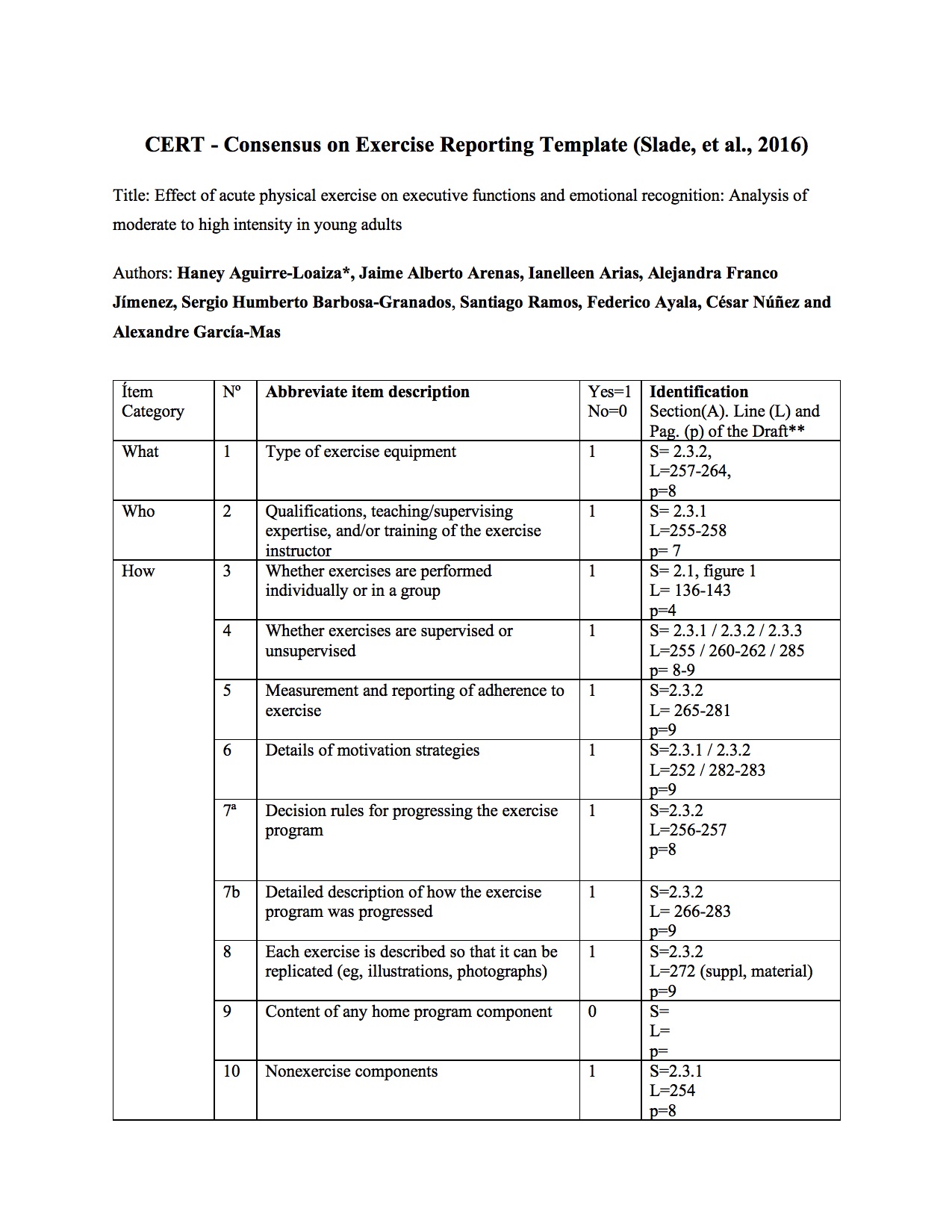

Supplement: TABLE S1 — Consensus on Exercise Reporting Template. [file Image_2.JPEG]
